# Comparison of three methods for detection of gametocytes in Melanesian children treated for uncomplicated malaria

**DOI:** 10.1186/1475-2875-13-319

**Published:** 2014-08-14

**Authors:** Stephan Karl, Moses Laman, Tamarah Koleala, Clemencia Ibam, Bernadine Kasian, Nola N’Drewei, Anna Rosanas-Urgell, Brioni R Moore, Andreea Waltmann, Cristian Koepfli, Peter M Siba, Inoni Betuela, Robert C Woodward, Timothy G St Pierre, Ivo Mueller, Timothy ME Davis

**Affiliations:** School of Medicine and Pharmacology, University of Western Australia, Fremantle Hospital, Fremantle, Western Australia Australia; School of Physics, University of Western Australia, Fremantle Hospital, Fremantle, Western Australia Australia; Infection and Immunity Division, Walter and Eliza Hall Institute, 1G Royal Parade, Parkville, 3052 Victoria Australia; Department of Medical Biology, The University of Melbourne, Melbourne, Victoria Australia; Papua New Guinea Institute of Medical Research, Madang, Madang Province Papua New Guinea; Center de Recerca en Salut Internacional de Barcelona (CRESIB), Barcelona, Spain; Institute of Tropical Medicine Antwerp, Antwerp, Belgium

**Keywords:** Malaria, Gametocyte detection, Magnetic fractionation, Reverse transcriptase PCR, Light microscopy *Plasmodium falciparum*, *Plasmodium vivax*, Papua New Guinea

## Abstract

**Background:**

Gametocytes are the transmission stages of *Plasmodium* parasites, the causative agents of malaria. As their density in the human host is typically low, they are often undetected by conventional light microscopy. Furthermore, application of RNA-based molecular detection methods for gametocyte detection remains challenging in remote field settings. In the present study, a detailed comparison of three methods, namely light microscopy, magnetic fractionation and reverse transcriptase polymerase chain reaction for detection of *Plasmodium falciparum* and *Plasmodium vivax* gametocytes was conducted.

**Methods:**

Peripheral blood samples from 70 children aged 0.5 to five years with uncomplicated malaria who were treated with either artemether-lumefantrine or artemisinin-naphthoquine were collected from two health facilities on the north coast of Papua New Guinea. The samples were taken prior to treatment (day 0) and at pre-specified intervals during follow-up. Gametocytes were measured in each sample by three methods: i) light microscopy (LM), ii) quantitative magnetic fractionation (MF) and, iii) reverse transcriptase PCR (RTPCR). Data were analysed using censored linear regression and Bland and Altman techniques.

**Results:**

MF and RTPCR were similarly sensitive and specific, and both were superior to LM. Overall, there were approximately 20% gametocyte-positive samples by LM, whereas gametocyte positivity by MF and RTPCR were both more than two-fold this level. In the subset of samples collected prior to treatment, 29% of children were positive by LM, and 85% were gametocyte positive by MF and RTPCR, respectively.

**Conclusions:**

The present study represents the first direct comparison of standard LM, MF and RTPCR for gametocyte detection in field isolates. It provides strong evidence that MF is superior to LM and can be used to detect gametocytaemic patients under field conditions with similar sensitivity and specificity as RTPCR.

**Electronic supplementary material:**

The online version of this article (doi:10.1186/1475-2875-13-319) contains supplementary material, which is available to authorized users.

## Background

The development of *Plasmodium* parasites within infected erythrocytes can follow one of two pathways: i) asexual reproduction for propagation within the human host or, ii) development of sexual reproductive blood stages (gametocytes) which can infect the mosquito vector when taken up with a blood meal. While asexual reproduction causes the clinical symptoms associated with malaria, sexual reproduction is responsible for inter-host spread of the parasite [1]. Gametocytes have many characteristics that differentiate them from asexual forms including, in the case of *Plasmodium falciparum*, a much longer life span and relative metabolic inactivity during their mature phase, which means that they are relatively resistant to anti-malarial drugs [2–4]. Artemisinin combination therapy (ACT) is, however, more effective against *P. falciparum* gametocytes than conventional agents, such as chloroquine or amodiaquine [5–8]. This increased effectiveness largely stems from the ability of ACT to destroy a wider range of early stage gametocytes, presumably at their sequestration sites [5, 9].

Monitoring gametocyte density in clinical isolates is necessary to assess the transmission-reducing effects of anti-malarial drugs or in investigations aiming to quantify the transmission potential of humans living in endemic areas [2, 10]. On-site detection of gametocytes at health facilities in malaria-endemic developing countries would also be beneficial when primaquine is being used to reduce *P. falciparum* gametocyte prevalence and thus transmission potential. Primaquine has potential toxicity, mainly haemolysis in G6PD-deficient individuals, and its use should ideally be limited to confirmed *P. falciparum* gametocyte carriers [2].

There is, therefore, a compelling argument for clinical and epidemiological studies to include sensitive and reliable methods for the detection of gametocytes. Gametocyte density in infected individuals is often too low to be detected by standard light microscopy (LM) [11] and a variety of molecular methods have been developed for gametocyte detection. These include reverse transcriptase polymerase chain reaction (RTPCR), quantitative nucleic acid sequence based analysis (QTNASBA) and reverse transcriptase loop mediated amplification (RTLAMP) [4, 12–16]. These methods usually target transcripts of the *P. falciparum Pfs25* gene or its homologue in *Plasmodium vivax* (*Pvs25*). However, molecular methods for gametocyte detection are not easily transferable to remote field laboratories or health facilities, are prone to contamination, use expensive equipment and reagents, and require highly trained personnel.

The aim of the present study was to assess the performance of a previously developed method for enhanced detection of gametocytes using quantitative high field gradient magnetic fractionation (MF) under field conditions in comparison to LM and RTPCR using samples collected during an anti-malarial clinical trial [17–19].

## Methods

### Background methodological considerations

It should be noted that the three methods employed in the present study quantify gametocytes in different ways:

i) LM quantifies the number of gametocytes per white blood cell (WBCs) (in the present study in 500 WBC) and extrapolates this value to calculate a gametocyte density per μL based on the assumption that 1 μL of blood contains 8,000 WBCs. The inherent threshold of detection of LM is therefore 16 gametocytes per μL of blood [18, 20];
ii) RTPCR uses *Pfs25* or *Pvs25* copy number as a measure for gametocyte abundance. As the number of these transcripts gradually increases while gametocytes maturate, this estimate may be different from LM-derived gametocyte counts, based on the stage distribution gametocytes in a blood sample. Although previous studies have derived calibration curves that related *Pfs25* abundance with mature stage V gametocyte density, it can be expected that field-based estimates vary considerably since blood samples may contain a wider range of gametocyte stages [21]. Assuming ideal performance, the detection threshold of RTPCR is only limited by the amount of blood subjected to amplification. In the present study, RNA was extracted from the equivalent of 50 μL of blood and eluted in 30 μL of RNAse free water. Four μL of this elution were then subjected to the amplification reaction. Therefore an equivalent volume of 6.7 μL of blood was tested by RTPCR, giving a theoretical detection limit of 0.13 gametocytes per μL of blood;
iii) MF uses an internal standard (magnetic microparticles), which is added to the sample at a known concentration per μL of blood so that the number of gametocytes counted per microparticle may be extrapolated to a gametocyte density per μL of blood. In the present study this known microparticle concentration was 100 μL and the maximum number of particles counted was 2,000 so that the expected detection threshold of MF was 0.05 gametocytes per μL of blood.

The conversion from one of these measures to another is not always straightforward, particularly in field samples containing different stages of gametocytes that may exhibit different levels of transcript expression and/or different magnetic susceptibilities. Furthermore, microscopic parasite and gametocyte counts as well as PCR methods are associated with considerable error [22, 23]. Therefore, field-based estimates for conversion factors between gametocyte quantification techniques are also likely to be associated with significant degrees of uncertainty.

### Sample collection and study site

This study received ethical approval as part of a larger clinical trial (Australian New Zealand Clinical Trials Registry ACTRN12610000913077) assessing the effect of recommended and novel artemisinin combination therapy in children with uncomplicated malaria from Madang Province in Papua New Guinea (PNG) (Medical Research Advisory Committee, Health Department of Papua New Guinea, approval 10.39). All research conducted on humans complied with the Helsinki declariation. Blood samples were collected at Mugil (n = 62) and Alexishafen (n = 8) Health Centres on the north coast of PNG near the town of Madang. Participants were enrolled after informed consent had been obtained. Children with clinical or laboratory evidence of severe malaria according to WHO severity criteria [24] or with co-morbidities were excluded.

Participating children were aged between 0.5 and five years and presented either with uncomplicated *P. falciparum* (minimum parasite density 1,000 μL^-1^) or *P. vivax* (minimum parasite density 250 μL^-1^) malaria. A total of 70 children were enrolled into this study (56 with *P. falciparum*, 14 with *P. vivax*). After recruitment, all participants were randomly assigned to receive treatment with either artemether-lumefantrine (1.7:10 mg/kg twice daily for three days) or artemisinin-naphthoquine (20:8 mg/kg daily for three days). For LM and MF, blood samples (200 μL) were collected on the day of enrolment (day 0) and on days 1, 2, 3, 7, 14, 28, and 42. For RTPCR additional blood samples (200 μL) were collected on days 0, 3, 7, and 28. After collection, these samples were directly transferred into a 1.5 mL tube containing the RNA preservation reagent RNAlater® (Life Technologies, Mulgrave, VIC, Australia).

### Gametocyte detection techniques

i) LM*:* Giemsa-stained thick blood smears were examined and parasite density quantified independently by two skilled microscopists with parasites counted against 500 white cells. Discrepancies were adjudicated by a senior microscopist.
ii) MF: Diluted whole blood was subjected to high field gradient MF as described elsewhere but a 200 μL volume was used instead of 400 μL as in previous studies. [17, 18]. Briefly, the 200 μL blood samples were suspended in 2 mL of MF buffer (PBS pH = 7.4, 0.5% bovine serum albumin, 2 mM EDTA). Magnetic particles (Spherotech, Lake Forest, IL, USA) were added to achieve a total known particle concentration of 100 particles per μL of blood. The samples were then magnetically fractionated as previously described using MACS equipment (Miltenyi Biotec, Bergisch Gladbach, Germany) [17] After passage of each sample through the columns, the columns were washed twice with 1 mL of MF buffer. Samples were then eluted in 1 mL of MF buffer and spun briefly to pellet the eluted material (cells/particles). Slides were prepared using the entire pellet. Slides prepared after MF were examined by screening the entire slide for gametocytes. If a gametocyte was observed, gametocytaemia was quantitatively assessed following the methodology described in [17] with slight modifications, and by counting a maximum number of 2,000 magnetic particles and the corresponding number of gametocytes in the associated fields. It should be noted that it takes only approximately 5 min to scan every field on an MF slide for gametocytes using 1,000× magnification, as the preparations contained very few cells.
iii) RTPCR: Samples collected in the RNA preservation reagent were transferred to a -80°C freezer as soon as possible (usually on the same day) after collection. RNA was extracted from the preserved samples using the Qiagen RNeasy 96 Plus kit (Qiagen, Doncaster, VIC, Australia) following manufacturer’s instructions with slight modifications. The sample volume subjected to extraction was equivalent to 50 μL of blood. Extracted RNA was eluted into 30 μL of RNAse free water. Four μL from the elution were subjected to the RTPCR reaction (equivalent of 6.7 μL of the original blood sample). Details of the RTPCR assay, including mix preparation, primer and probe sequences have been published elsewhere [21]. Each detection experiment included a duplicate dilution series of plasmids containing the Pfs25 or Pvs25 target product (104, 103, 102, 101, 5, 100 copies/μL) that was used to determine standard curves and estimate gametocyte densities reported as transcript copies/μL. All assays were run in 384-well plate format on the Roche LightCycler480® platform.

### Data analysis

Data were compared between LM *vs* MF, LM *vs* RTPCR and MF *vs* RTPCR for the number of sample pairs available in each of these three groups. Proportions were compared by McNemar’s exact test. Sensitivity and specificity were calculated from the numbers of true/false positives and negatives when each of the methods was considered to be the reference method. Quantification of gametocytes by each method was compared using correlation analyses (Pearson), censored regression analyses (Tobit regression [25]) and Bland-Altman analyses. Since the decadic logarithms of the estimated gametocyte densities/transcript copy numbers were normally distributed (confirmed by Kolmogorov-Smirnov tests), these transformations were used in the analyses.

## Results

### Observed gametocyte positivity

Paired observations (LM *vs* MF) were available for 491 blood samples (402 originating from *P. falciparum-*infected children and 89 from *P. vivax*-infected children). From the subset of samples subjected to RTPCR, 238 paired observations for LM *vs* RTPCR (195 from *P. falciparum-* and 43 from *P. vivax-*infected children) and 251 paired observations for MF *vs* RTPCR (204 from *P. falciparum-* and 47 from *P. vivax-*infected children) were available. Since *P. vivax* gametocytes are cleared rapidly after treatment, the number of *P. vivax*-positive paired observations were very limited in the present study.

**Figure 1:**
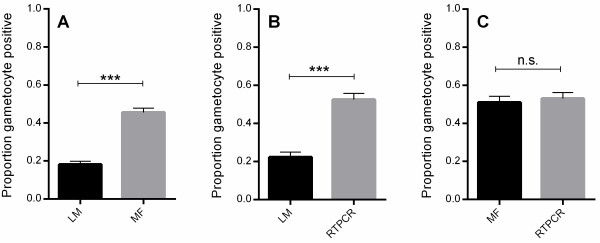
**Proportion of gametocyte positive observations using LM** ***vs.*** **MF.** (Panel **A**), LM *vs.* RTPCR (Panel **B**) and MF *vs.* RTPCR (Panel **C**).The analysis is purely concerned with the performance of the three methods and does not take into account the drug used for treatment or time the sample was taken. (n.s.: not significant; ***p-value <0.001 (McNemar’s test).

Figure 1 shows the proportions of gametocyte-positive observations for the three methods. Overall, LM resulted in approximately 20% gametocyte-positive observations, whereas MF and RTPCR both resulted in more than two-fold higher observed gametocyte positivity (LM *vs* MF: 18.1 *vs* 45.6%, p < 0.0001; LM *vs* RTPCR: 22.2 *vs* 52.5%, p < 0.0001; MF *vs* RTPCR: 51.0 *vs* 53.0%, p = 0.61). As most blood samples originated from children who had already received anti-malarial treatment, the proportions displayed in Figure 1 do not reflect the gametocyte positivity rate in the population but should be viewed as a measure of how well the three methods were able to detect gametocytes. At time of enrolment (prior to treatment) observed gametocyte prevalence using LM was around 29% (20/70), whereas with both MF and RTPCR an approximate gametocyte positivity of 86% (60/70 for MF and RTPCR) was found.

The comparative sensitivity and specificity of each of the three gametocyte-detection methods are presented in Table 1. Within each comparison, each method was considered as the reference method (e.g., LM is compared to MF once with LM as reference method and once with MF as reference method). This is necessary since LM is still considered the ‘gold standard’, yet its sensitivity for gametocyte detection is inferior to both MF and RTPCR methods, and analyses of specificity are not meaningful if LM is used as the only reference method as the number of ‘false positives’ will be biased.

**Table 1:** **Comparison of sensitivity and specificity of gametocyte detection between light microscopy (LM), magnetic fractionation (MF) and reverse transcriptase polymerase chain reaction (RTPCR)**

A1		LM		Total	A2	Reference	
		0	1			LM	MF
MF	0	266	1	267	Sensitivity	0.99	0.39
	1	136	88	224	Specificity	0.66	0.99
Total		402	89	491			
**B1**		**LM**		**Total**	**B2**	**Reference**	
		**0**	**1**			**LM**	**RTPCR**
RTPCR	0	110	3	113	Sensitivity	0.94	0.40
	1	75	50	125	Specificity	0.59	0.97
Total		185	53	238			
**C1**		**RTPCR**		**Total**	**C2**	**Reference**	
		**0**	**1**			**RTPCR**	**MF**
MF	0	89	34	123	Sensitivity	0.74	0.77
	1	29	99	128	Specificity	0.75	0.72
Total		118	133	251			

In each of the three comparisons (A: LM *vs* MF; B: LM *vs* RTPCR and C: RTPCR *vs* MF) both methods served as reference standard.

When LM is compared to MF with LM as the reference method, sensitivity is very high (99%), indicating that MF detects nearly all positive cases detected by LM. Specificity however is low, since LM is inferior to MF in sensitivity to detect gametocytes and the analysis thus results in a high number of ‘false positive’ observations. When MF is used as the reference method in this comparison LM has a very low sensitivity (39%). The specificity is very high (99%) indicating that MF indeed identifies nearly all gametocyte carriers as correctly as LM.

The situation is similar when LM is compared to RTPCR. Again, with LM as the reference method, RTPCR sensitivity is high (94%) and specificity is artificially low. When RTPCR is considered the reference standard, LM sensitivity is low (40%) whereas specificity is high (97%) indicating that RTPCR identifies most gametocyte carriers correctly.

When MF is compared to RTPCR, interchanging the reference methods has little effect. This indicates that both of these methods are similarly sensitive and specific for gametocyte detection, although they only agree with each other moderately well (~75%).

### Observed gametocyte densities

**Figure 2:**
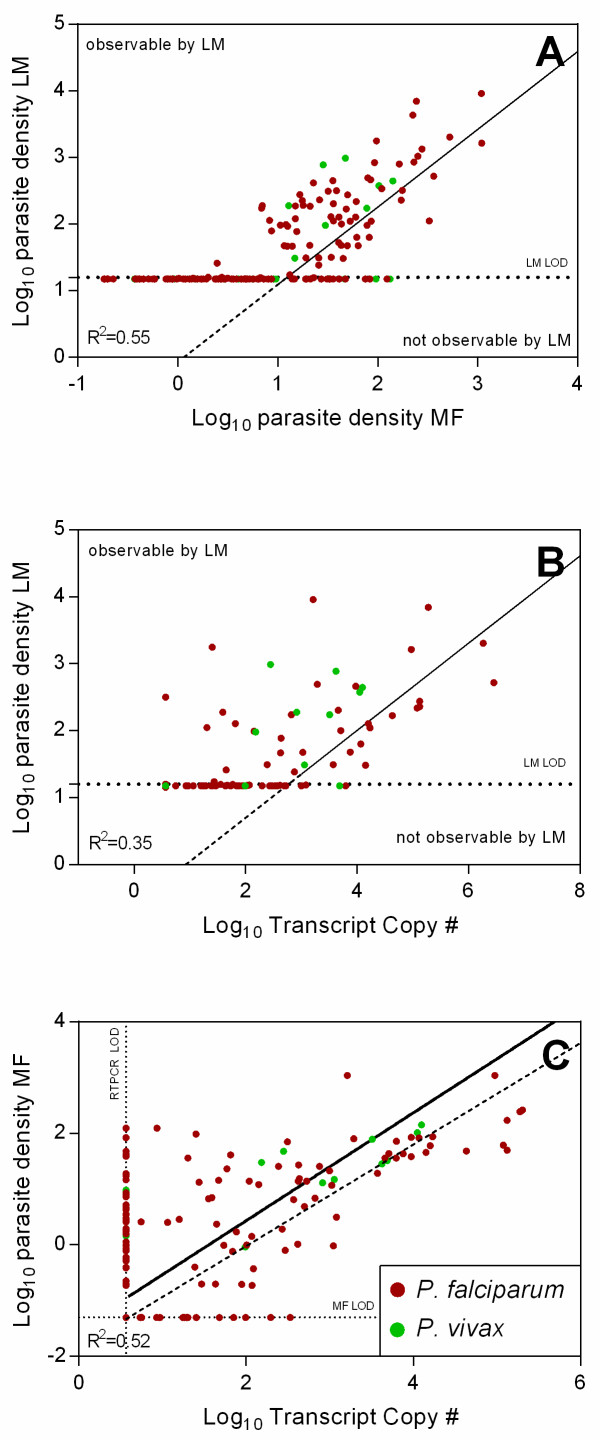
**Relationship between gametocyte abundance measured by LM, MF and RTPCR.** Panel **A**: LM gametocyte density is plotted *vs* MF gametocyte density. The data are heavily left-censored since many observations cluster at the detection limit of LM. Therefore, a censored regression (Tobit model [25]) was used instead of standard linear regression, resulting in the depicted regression line. Panel **B**: LM gametocyte density is plotted *vs Pfs25* and *Pvs25* copy numbers. The black line is the best fit curve given by the Tobit regression. Panel **C**: MF gametocyte density is plotted over RTPCR transcript copy number. The continuous black line is the best fit by orthogonal regression. The dashed black line is the reference estimate from serially diluted strain 3D7 gametocyte cultures published by Wampfler *et al.*[21].

LM reports gametocyte density based on gametocyte counts relative to leukocyte counts; MF uses an internal standard in the form of magnetic microspheres [17]; RTPCR derives *Pfs25* or *Pvs25* copy numbers from a standard curve. Figure 2 shows the gametocyte abundance observed with the three methods (LM, RTPCR and MF). The correlations between these measures are highly significant (Pearson p < 0.0001). LM gametocyte density estimates ranged from 16 and 9200 μL^-1^ for *P. falciparum* and between 16 and 780 μL^-1^ for *P. vivax*.

When comparing the more sensitive methods (MF and RTPCR) with LM, the data are heavily left-censored and clustered on the LM limit of detection (LOD) which is estimated to be 16 μL^-1^. Therefore, a censored linear regression model (Tobit model) was chosen for regression between LM and MF, and LM and RTPCR. The coefficients of determination (R^2^) from the Tobit regression were 0.55 and 0.35 for LM *vs* MF and LM *vs* RTPCR, respectively, implying only moderate degrees of predictability. For comparison of MF and RTPCR, which are both similarly sensitive, orthogonal linear regression was used and R^2^ was 0.52.

MF gametocyte density estimates were also used to derive a calibration curve between *Pfs25/Pvs25* transcript copy number and gametocyte number (Figure 2C). The resulting equation to estimate gametocyte density (y) from transcript copy number (x) was log_10_*y = 0.97log*_*10*_*x-1.51.* In the epidemiologically relevant range (i.e., > 0.1 gametocytes per μL) of blood this corresponds to around 30–40 transcripts per gametocyte. This estimate of transcripts per gametocyte is lower than the one previously derived from serially diluted gametocyte cultures (~70-110 transcripts per gametocyte) [21].

**Figure 3:**
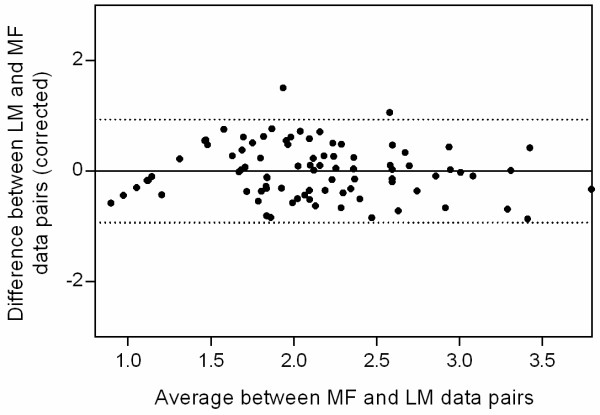
**Corrected Bland and Altman comparison between LM and MF.** The 95% confidence levels of agreement were +/-0.93 (dotted lines in the graph).

Since LM and MF both determine a gametocyte density (LM based on a leukocyte count and MF based on a count of gametocytes *vs* magnetic particles), the Bland and Altman method comparison was used to compare these two methods (Figure 2, Panel A) for those data pairs that were not left-censored. [26] Bland and Altman analysis showed no significant trends in the observed differences between LM and MF over average gametocyte density (Additional file 1), however it revealed that the logarithms of the MF-based measures of gametocyte density were biased consistently with a mean of -0.59 against the LM estimates. Therefore, to transform MF gametocyte density into LM gametocyte density, this bias was taken into account by adding 0.59 to each logarithmic MF gametocyte density. The resulting corrected 95% confidence levels of agreement between LM and MF were +/-0.93, meaning that MF and LM estimates of gametocyte density can be expected to vary not more than approximately ± ten-fold in 95% of measurements (Figure 3).

## Discussion

The aim of the present study was to assess the performance of MF compared to that of standard LM and RTPCR for *Pfs25* and *Pvs25* for gametocyte detection in a field study. Based on previous research, it was hypothesized that MF would generate highly sensitive quantitative gametocyte measurements under field conditions. MF showed an at least similar ability to detect gametocyte carriers as the RTPCR method, and both MF and RTPCR detected significantly more gametocyte-positive samples than LM. Measures of gametocyte density were strongly associated between methods and conversions between them could be derived. However, these conversions are associated with considerable levels of uncertainty.

The results presented here show that MF was similarly sensitive in detecting gametocytes as RTPCR conducted on blood samples directly placed into an RNA preservation solution at the time of collection. While both MF and RTPCR detected many (at least two to three-fold) more gametocyte-positive samples than standard LM, there was no significant difference in the proportions of gametocyte-positive samples detected by MF and RTPCR (Figure 1) indicating equivalent performance. Both MF and RTPCR showed very high sensitivity (99 and 94%, respectively) and specificity (99 and 97%, respectively) for gametocyte detection when compared with LM. When compared to RTPCR, MF showed similar sensitivity and specificity.

In terms of quantification, MF produced results that were in agreement with LM observations (Figure 2A). However, directly calculated gametocyte densities based on the number of gametocytes per magnetic particle were consistently lower than the gametocyte density estimates by LM. Since this phenomenon was systematic across the entire range of observed gametocyte densities (Figure S1), a simple correction for this negative bias could be applied resulting in a good agreement between LM gametocyte densities and the corrected MF derived gametocyte densities. There are several possible explanations for this observed bias. Firstly, it has been shown that the assumption of 8,000 WBCs per μL of blood may lead to an overestimate of parasite density (if individuals have lower WBC counts) [27]. However, automated WBC counts from children in the present study did not indicate large deviation from the assumed 8,000 WBC per μL [28]. Another possible explanation is the differential magnetic susceptibility between magnetic particles and gametocytes. The magnetic particles used in the present study had a significantly higher magnetic susceptibility compared to gametocytes which may lead to a differential capture efficiency of magnetic particles and gametocytes in the MF columns. Previous studies have shown that the capture efficiency for gametocytes by MF is approximately 50% [29]. This may lead to a consistent underestimation of gametocyte density by MF. The usage of magnetic particles that have the same magnetic susceptibility as gametocytes may reduce this effect.

MF estimates of gametocyte densities were used to derive a log-linear equation to convert transcript copy number of *Pfs25* or *Pvs25* into gametocyte densities. The resulting estimates for the number of transcripts per gametocyte are lower than that in previous studies (less transcripts per gametocyte, Figure 2C), which may be due to several reasons: i) although the ratio of blood to RNA preservation reagent and ultimately, the blood volume subjected to the RTPCR reaction, was very similar to that used in the study by Wampfler *et al*. [21], even slight differences in the extraction and amplification protocols may result in considerable differences in the observed transcript copy numbers; ii) although the samples were placed in RNA preservation reagent, several hours could pass before these samples were transferred from the field to the laboratory and for frozen storage and this may have led to some RNA degradation [30]; and, iii) while Wampfler *et al*. [21] derived the standard curve using serially diluted laboratory-cultured gametocytes that were synchronous stage V, the estimate in the present study is based on much more variable data (field based gametocyte counts including all identifiable gametocyte maturation stages) so that a lower estimated transcript copy number per gametocyte is expectable.

The present study shows that MF can be a valuable tool in determining gametocyte positivity and density under field conditions in malaria studies. Although MF is slower when conducted on batches of samples, it has the advantage over RTPCR that it can be directly applied in the field without much additional equipment or expertise and without concerns regarding contamination or appropriate sample storage. Direct identification of gametocyte carriers, is important if targeted primaquine treatment of *P. falciparum* gametocyte carriers is considered to reduce transmission of *P. falciparum*. In order to minimize the risk of primaquine toxicity, MF could be used to confirm gametocyte carriage even if the health centre is remote and with limited electricity [5, 31, 32]. Furthermore, since symptomatic vivax malaria is less frequent in countries such as PNG, MF can be used to improve recruitment rates of eligible patients with gametocytes into epidemiological follow-up studies or primaquine dose escalating trials aiming to identify optimal dosing schedules.

The present study provides evidence that MF is a feasible methodology for field-based gametocyte detection that can be applied even in larger scale studies. With an appropriate set-up, four to six samples could conveniently be processed by one person in an hour with results being available for all samples after approximately 1.5 hr.

## Conclusions

The present study shows that magnetic fractionation is a very good alternative method for on-site gametocyte detection under field conditions. The method has, as previously shown under laboratory conditions, similar sensitivity to RTPCR and can be used to derive quantitative gametocyte densities for both *P. falciparum* and *P. vivax*.

## Electronic supplementary material

Additional file 1: **Bland and Altman comparison between LM and MF.** A consistent negative bias of -0.59 between MF and LM was observed and corrected for (shown in Figure 3 in the main text). The red horizontal line denotes ‘0 bias’. (TIFF 21 KB)
